# KS-cSCC-1 and KS-cSCC-2: two novel cutaneous squamous cell carcinoma cell lines established from Japanese patients

**DOI:** 10.3389/fmed.2024.1483450

**Published:** 2024-11-07

**Authors:** Takamichi Ito, Yuka Tanaka, Yumiko Kaku-Ito, Keiko Tanegashima, Mao Imajima, Toshio Ichiki, Takeshi Nakahara

**Affiliations:** Department of Dermatology, Graduate School of Medical Sciences, Kyushu University, Fukuoka, Japan

**Keywords:** cutaneous squamous cell carcinoma, cancer cell line, chemosensitivity, immunohistochemistry, cell line establishment

## Abstract

**Introduction:**

Cutaneous squamous cell carcinoma (cSCC) is a common form of skin cancer. Less accessibility to the cSCC cell lines has limited analyses of this disease. Thus, we here aimed to establish novel cSCC cell lines from patient's cSCC lesions.

**Methods:**

Two novel cSCC cell lines (named KS-cSCC-1 and KS-cSCC-2) were established from an axillary lymph node metastasis of a Japanese female and an inguinal lymph node metastasis of a Japanese male. The characteristics of the established cell lines were assessed by *in vitro* analyses.

**Results:**

The cells were successfully maintained for more than 9 months, with a doubling time of 47.5 ± 1.11 h (KS-cSCC-1) and 39.2 ± 5.78 h (KS-cSCC-2). The cell lines exhibited constant growth, spheroid formation, and invasiveness. Short tandem repeat analyses and immunohistochemistry confirmed that both cell lines are identical to their original tumor. The KS-cSCC-1 cells were weakly positive for CK14 and strongly positive for CK10, while the KS-cSCC-2 showed opposite expression patterns. Chemosensitivity of the cell lines was further tested and the cells were sensitive to anticancer drugs which are used to treat cSCC.

**Conclusion:**

The KS-cSCC-1 and KS-cSCC-2 cell lines were promising resources for basic and preclinical research on cSCC to better define the tumor characteristics and treatment strategy of this cancer.

## 1 Introduction

Cutaneous squamous cell carcinoma (cSCC) is a common form of skin cancer that is characterized by the malignant growth of epidermal keratinocytes. It is classified as a keratinocyte carcinoma along with basal cell carcinoma (1, 2). cSCC is the second most prevalent type of skin cancer, accounting for ~20% of all skin cancers (1–4). The majority of primary cSCCs (80%−90%) are located on the head and neck (5). The incidence of cSCC is increasing worldwide (4, 6–9). Primary cSCCs are typically slow-growing and can be cured with appropriate treatment. The 5-year cure rate for cSCC is over 90% and the estimated rate of local recurrence is 2%−5% (1, 10–12).

Surgical removal of the tumor is the primary treatment for cSCC. However, certain high-risk features such as large size, location on lip/ear/temple, deep invasion, poor differentiation grade, presence of desmoplasia, perineural invasion, bone erosion, immunosuppression, and positive surgical margins may cause recurrence or metastasis. Around 2%−5% of cSCCs metastasize to lymph nodes or distant sites, leading to an unfavorable prognosis (13, 14). Intensive efforts have been made to better understand the etiology of cSCC and establish an effective therapy for metastatic cSCC, including immunotherapy, molecular-targeted therapy, cytotoxic chemotherapy, radiotherapy, and their combinations (15–33). Recent systematic reviews have suggested anti-PD-1 therapy to be the most promising treatment for advanced cSCC (34, 35). A pivotal clinical trial for cSCC using cemiplimab, an anti-PD-1 antibody, resulted in a response rate of 47.2%, with 17.1% being complete responses and 30.1% being partial responses. Additionally, the treatment was relatively well tolerated, with 10.4% of patients discontinuing treatment due to adverse events (36). Both the US Food and Drug Administration (FDA) and the European Medicines Agency have approved the use of cemiplimab for patients with locally advanced or metastatic cSCC who are not eligible for curative surgery or radiotherapy. Pembrolizumab, another anti-PD-1 antibody, has also been approved by the FDA for the treatment of cSCC (37). However, these therapies are not perfect solutions, and there is still a need for more effective treatments for patients with progressive disease who have already received anti-PD-1 therapy.

Cancer cell lines are an important tool for conducting basic and preclinical research, as they allow functional analyses. The A431 cell line is currently commercially available and is commonly used for the analysis of cSCC. However, this cell line was originally established from a vaginal cancer and should be distinguished from cSCC (38–40). In the current study, we successfully established two novel cSCC cell lines, named KS-cSCC-1 and KS-cSCC-2, from both a female and a male Japanese patient. These cell lines represent a promising resource for both basic and preclinical research.

## 2 Materials and methods

### 2.1 Ethical approval

This study was conducted in accordance with the tenets of the Declaration of Helsinki. The Ethics Committee of Kyushu University Hospital approved the study (Approval no. 21050-00, granted on November 10th, 2021). Prior to their inclusion in the study, the patients provided written informed consent.

### 2.2 Immunohistochemistry (IHC)

The staining methods described in previous reports were used to stain the patient's tissue (41–43). Formalin-fixed, paraffin-embedded tissues of cSCC were obtained from the archives of Kyushu University Hospital (Fukuoka, Japan). The primary antibodies used for staining were p63 (prediluted, 713751; Nichirei Biosciences Inc., Tokyo, Japan), AE1/AE3 (prediluted, 412811; Nichirei Biosciences Inc.), CK14 (1:100, ab7800; RRID: AB_306091, Abcam, Cambridge, UK), CK10 (1:50, M7002; DAKO, Glostrup, Denmark), CAM5.2 (prediluted, 349205; RRID: AB_2134314, Becton Dickinson, Franklin Lakes, NJ), and CEA (prediluted, 413121; Nichirei Biosciences Inc.). The secondary antibody used was *N*-Histofine Simple Stain MAX-PO MULTI (724152; Nichirei Biosciences Inc.). The sections were further reacted with 3,3′-diaminobenzidine tetrahydrochloride substrate (725191; Nichirei Biosciences Inc.) to detect signals and counter stained with hematoxylin (30002; Muto Pure Chemicals, Tokyo, Japan) to stain the nucleus. The stained samples were examined and captured using a Nikon ECLIPSE 80i microscope (Nikon, Tokyo, Japan).

### 2.3 Quantification of IHC

IHC for AE1/AE3, CD10, and CK14 were semi-quantitatively evaluated, using the H-score. In each high-power field (HPF, × 400), the positivity of each cell was classified into the following four groups; negative: 0, weakly positive: 1, moderately positive: 2, and strongly positive: 3. The H-score (0–300) was calculated by multiplying the proportion (%) of stained cells by the staining intensity (0–3). For bioimaging analysis, QuPath ver. 5.1 (44), an open-source platform, was used. Images were imported as JPEG images of the HPFs and positive cell detection to recognize the proportion and intensity of the IHC was performed. The nuclear parameters were set as follows: nuclear size of 10 to 1,500 pixels, cell expansion of 5 pixels, and background radius of 15 pixels. As for the intensity threshold parameters, the mean staining intensity in the cytoplasm was evaluated and divided into three threshold levels: weak (+1, highlighted in yellow), moderate (+2, orange), and strong (+3, red). Negatively stained cells were highlighted in blue. Then, QuPath software automatically provided an H-score for each image (Supplementary Figure 1).

### 2.4 Establishment and culture of patient tissue-derived cSCC cell lines

The patient tissue-derived cell lines were established as previously reported (45). The specimen was obtained from a 62-year-old female with a primary cSCC on the hand and axillary lymph node metastases, and an 81-year-old male with a primary cSCC on the thigh and inguinal lymph node metastases, both of whom were treated at the Department of Dermatology, Kyushu University Hospital (Fukuoka, Japan). A metastatic axillary lymph node of the 62-year-old female (KS-cSCC-1), and a metastatic inguinal lymph node of the 81-year-old male (KS-cSCC-2) was used for cell line establishment. The tissues were cut into small pieces using sterilized scissors and digested in DMEM (D6429; Sigma-Aldrich, St. Louis, MO) supplemented with 10% fetal bovine serum (FBS, 174012; Nichirei Biosciences Inc.), 1 mg/mL collagenase type I (031-17601; Fujifilm Wako Pure Chemicals, Osaka, Japan), and antibiotics (15240-062; Thermo Fisher Scientific, Waltham, MA) for 1 h at 37°C in water bath while vortexing every 10 min. Digested tissue was passed through a 100 μm cell strainer (352360; Corning, Corning, NY) and washed with DPBS (21600010; Thermo Fisher Scientific). The cells were then suspended in KGM-Gold Basal Medium with SingleQuots supplements (00192060; Lonza, Basel, Switzerland), seeded in a 100 mm culture dish coated with Cellmatrix Type I-A (Nitta Gelatin, Osaka, Japan), and cultured at 37°C in 5% CO_2_. Culture medium was refreshed every 2–3 day and cells were passaged by trypsinization at 80% confluence. Cells were maintained for more than 6 months (15 passages) before being used for the experiments. The established cell lines were confirmed to be mycoplasma-free using a CycleavePCR Mycoplasma Detection Kit (CY232; Takara Bio Inc., Tokyo, Japan).

### 2.5 Cell culture of normal human keratinocytes and a human SCC cell line A431

Normal human epidermal keratinocytes (NHEKs, lot number: 19TL254790, 18TL155114, and 0000674241) were cultured in KGM-Gold Basal Medium with SingleQuots supplements. A431 human SCC cell line was cultured in DMEM supplemented with 5% FBS. Culture medium was refreshed every 2–3 day and cells were passaged by trypsinization at 80% confluence.

### 2.6 Short tandem repeat (STR) analysis

To confirm the identity of the established cell lines, STR analysis was conducted as previously reported (45). STR analysis was performed using the KS-cSCC-1 cells at passage 30 (9 months) and KS-cSCC-2 cells at passage 29 (>9 months). The STR of the samples was considered identical if the match ratio exceeded 0.8 (46).

### 2.7 Cell proliferation assay and cell doubling time

Cells were seeded into 96-well plates (5,000 cells/well) and cultured at 37°C in 5% CO_2_ until their growth reached plateau. On each day, viable cells were quantified using Cell Counting Kit-8 (CCK-8, 343-07623; Dojindo, Kumamoto, Japan). Absorbance of the resultant solution at 450 nm was measured. Cell growth rate was calculated as ln(N_*t*_/N_0_) divided by time *t*. Doubling time was further calculated as ln (2) divided by growth rate (N_0_: cell number at time 0, N_*t*_: cell number at time *t*).

### 2.8 Spheroid formation assay

To evaluate spheroid formation, cells were seeded into 96-well ultra-low-attachment microplates (3,000 cells/well, 7007; Corning) and incubated at 37°C in 5% CO_2_. On day 3, spheroids were observed using a microscope (Nikon) and images of spheroids were captured by a camera system (Nikon). Experiments were repeated three times with 6 technical replicate wells. To quantitate the spheroids, the number of spheroids was counted from the captured images and the projected area (mm^2^) of each spheroid was measured by ImageJ software (National Institutes of Health, Bethesda, MD).

### 2.9 Invasion assay

Invasiveness of the established cell lines was evaluated using a CytoSelect 24-well cell invasion assay (CBA-110; Cell Biolabs Inc., San Diego, CA) as described in our previous report (45). Cells were suspended in serum- and cytokine-free medium at a concentration of 5 × 10^5^ cells/mL, and 300 μL of the suspension was added into the cell culture insert in the lower well containing complete media. After 48 h incubation at 37°C in 5% CO_2_, invaded cells were stained with dye, washed with deionized water, air-dried, and observed under a microscope (Nikon). Images of invaded cells were captured using a camera system (Nikon). The invaded cells were counted manually from the images and cell number per unit area (mm^2^) was calculated.

### 2.10 Immunocytochemistry

Immunocytochemistry was performed as previously reported (45). The primary antibodies used were as follows: CK10 (1:150, ab76318; RRID: AB_1523465, Abcam), CK14 (0.2 μg/mL, ab181595; RRID: AB_2811031, Abcam), and AE1/AE3 (1:100, GTX75521; RRID: AB_376859, GeneTex Inc., Irvine, CA). The secondary antibodies used were as follows: Alexa Fluor^®^488-conjugated goat anti-rabbit IgG (A11008; RRID: AB_143165, Thermo Fisher Scientific) and Alexa Fluor^®^488-conjugated goat anti-mouse IgG (A11001; RRID: AB_2534069, Thermo Fisher Scientific). Mean fluorescence intensities of each marker per cell were measured using ImageJ software. At least three images from independent areas were used for the analysis.

### 2.11 RNA extraction and quantitative reverse-transcription polymerase chain reaction (qRT-PCR)

RNA extraction, cDNA conversion, and subsequent qPCR were performed as reported previously (45). To normalize the expression of measured genes, β-actin (*ACTB*) was used as an internal control gene. The sequences of the primers used are summarized in Supplementary Table 1.

### 2.12 Western blotting

Western blotting was performed as reported previously (47). The primary antibodies used were as follows: rabbit anti-human CK10 (1:10,000, ab76318; RRID: AB_1523465, Abcam), rabbit anti-human CK14 (1:20,000, ab181595; RRID: AB_2811031, Abcam), and rabbit anti-human β-Actin (1:2,000, 4970; RRID: AB_2223172, Cell Signaling Technology, Danvers, MA). The secondary antibody was goat anti-rabbit IgG horseradish peroxidase (1:10,000, 7074; RRID: AB_2099233, Cell Signaling Technology). The resultant bands were detected with SuperSignal West Pico Chemiluminescent Substrate (34580; Thermo Fisher Scientific) and the ChemiDoc XRS Plus System (Bio-Rad Laboratories Inc.). The intensity of the detected bands was quantified using ImageJ software.

### 2.13 Chemosensitivity assay

Chemosensitivity of the established cell lines to cisplatin (033-20091), 5-fluorouracil (068-01401), docetaxel (047-31281), irinotecan (091-06651), and doxorubicin (040-21521; all purchased from Fujifilm Wako Pure Chemicals) was evaluated by CCK-8 assay. Cells were seeded into 96-well plates at 5,000 cells/well and pre-cultured at 37°C in 5% CO_2_. After 24 h, medium was replaced with fresh medium containing vehicle (0.1%, saline for cisplatin, DMSO for 5-fluorouracil and docetaxel, distilled, culture-grade pure water for irinotecan and doxorubicin) or various concentrations of the drugs (0.01 nM to 100 μM at final concentration) and the cells were further incubated for 72 h at 37°C in 5% CO_2_. After incubation, the cells were treated with CCK-8 solution for 2 h and absorbance of the resultant solution at 450 nm was measured. IC_50_ of each drug was then calculated using GraphPad Prism 7 software (GraphPad Software, San Diego, CA).

### 2.14 Statistical analysis

All experiments were repeated at least three times independently, and results are displayed as the mean ± standard deviation. Statistical analyses were then performed using GraphPad Prism 7 software. The normality of the data distribution was determined using the Shapiro–Wilk test and the significance of differences between two groups was tested by Student's unpaired two-tailed *t*-tests. When the data were not normally distributed, Mann–Whitney U test was used. *p* < 0.05 was considered statistically significant.

## 3 Results

### 3.1 Histopathological features of the original cSCC lesions

**Figure 1:**
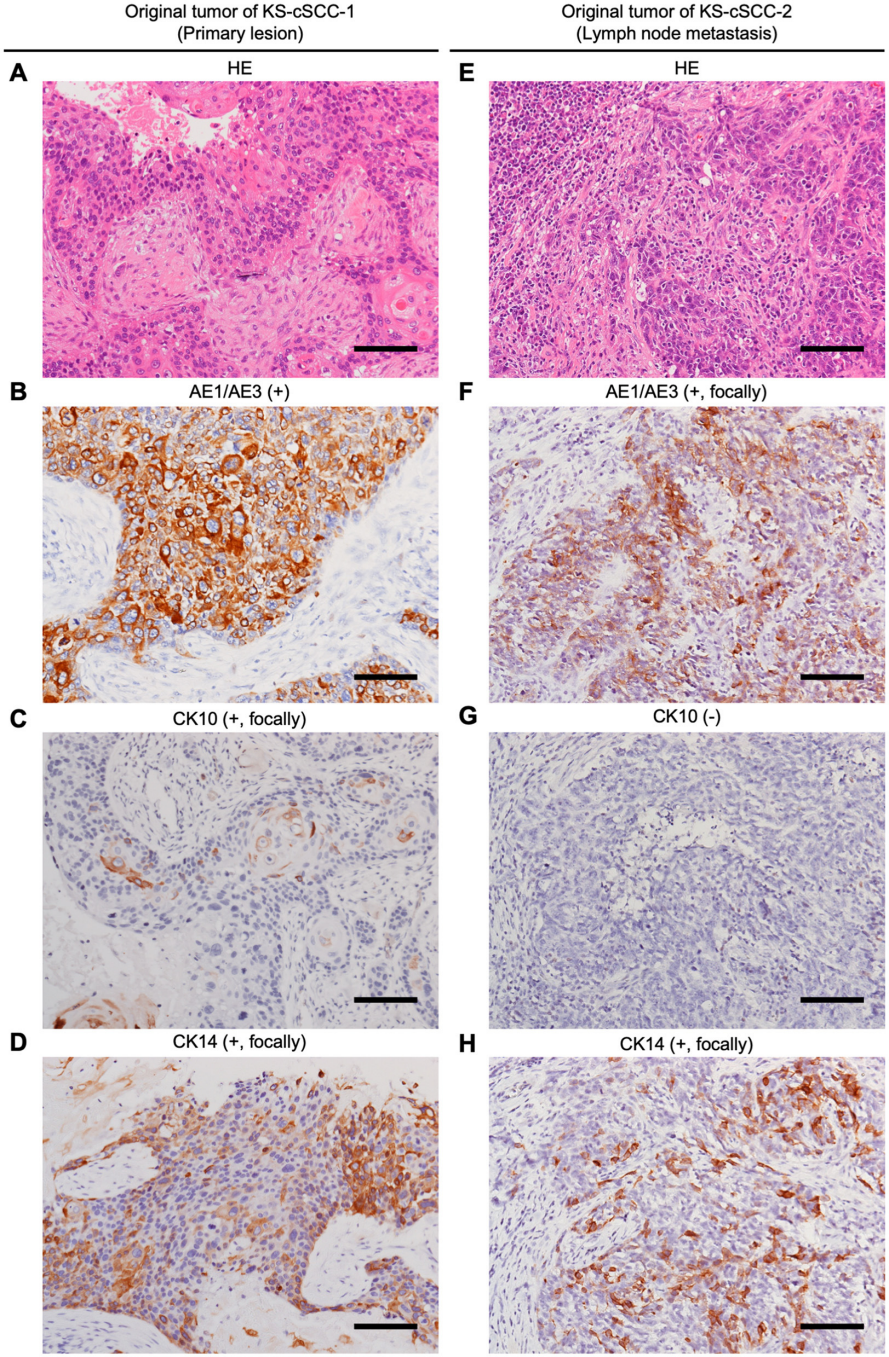
Histopathological analysis of the patient's cSCC tissues. **(A–D)** Histopathological images of original tumor tissue of KS-cSCC-1. **(A)** Hematoxylin and eosin (HE) staining of the metastasis showed massive nests of atypical keratinocytes with keratinous materials. **(B–D)** Immunohistochemically, the tumor cells were positive for AE1/AE3, focally positive for CK10 and CK14. **(E–H)** Histopathological images of original tumor tissue of KS-cSCC-2. **(E)** HE staining of the metastasis showed poorly differentiated tumor nests composed of atypical polygonal cells. **(F–H)** Immunohistochemically, the tumor cells were focally positive for AE1/AE3 and CK14, and negative for CK10. Scale bars = 100 μm.

Hematoxylin and eosin (HE) staining of the original metastatic tumor lesion of KS-cSCC-1 revealed massive nests of atypical keratinocytes with keratinous materials (Figure 1A). Abundant necrotic tissues were also observed. Immunohistochemically, the tumor cells were positive for AE1/AE3 (Figure 1B) and p63, focally positive for CK10 (Figure 1C) and CK14 (Figure 1D), and negative for CAM5.2 and CEA (data not shown), findings which were consistent with cSCC. Regarding the original metastatic tumor lesion of KS-cSCC-2, sheet-like proliferation of poorly differentiated atypical polygonal cells was found in HE staining (Figure 1E). Immunohistochemically, the tumor cells were positive for p63 (data not shown), focally positive for AE1/AE3 (Figure 1F), CK14 (Figure 1G) and CAM5.2, and negative for CK10 (Figure 1H) and CEA. The expressions of AE1/AE3, CK10, and CK14 were further compared in normal skin and primary tumor (Supplementary Figures 1, 2). All three markers were highly expressed in the normal tissue and tended to be decreased in primary tumors. The H-score of AE1/AE3, CK10, and CK14 of normal skin and primary tumor of KS-cSCC-1 was 201 and 240, 113 and 11.4, and 140 and 142, respectively. AE1/AE3, CK10, and CK14 of normal skin and primary tumor of KS-cSCC-2 was 271 and 109, 155 and 0.05, and 281 and 223, respectively. When compare the IHC staining of primary tumors of KS-cSCC-1 and KS-cSCC-2, AE1/AE3 was positive in primary tumor of KS-cSCC-1 (H-score of 240) and focally positive in primary tumor of KS-cSCC-2 (H-score of 109). CK10 was focally positive in the primary tumor of KS-cSCC-1 (H-score of 11.4) and very faint and focally positive in the primary tumor of KS-cSCC-2 (H-score of 0.05). CK14 was focally positive in primary tumors of both patients (H-score of 142 and 223 in KS-cSCC-1 and KS-cSCC-2, respectively). When compared with the established cell line, AE1/AE3 was slightly more highly expressed in KS-cSCC-2 than in KS-cSCC-1, opposite pattern observed in primary tumors. The expression pattern of CK10 and CK14 was the same in the cell lines and in primary tumors.

### 3.2 STR analysis

Two novel cSCC cell lines, KS-cSCC-1 and KS-cSCC-2, were established from metastatic lymph nodes that were surgically resected from both a female and a male Japanese cSCC patient. The cell lines were successfully maintained for >9 months (over 30 passages) *in vitro*. To investigate whether the genomes of the original cSCC lesions and the established cell lines are identical, STR analyses were performed. The allele data from the STR analysis are shown in Table 1. The STR match ratio was calculated as 0.88 and 0.93 for KS-cSCC-1 and KS-cSCC-2, respectively, indicating that the established cell lines have genomic features identical to the original cSCC lesions.

**Table 1:** Short tandem repeat analysis.

**Locus**	**Original cSCC lesion**		**KS-cSCC-1**	
TH01	6	9	6	
D21S11	28	30	28	30
D5S818	11	12	12	
D13S317	11	12	11	
D7S820	10	13	10	13
D16S539	9	11	9	11
CSF1PO	10	12	12	
AMEL	X		X	
vWA	18		18	
TPOX	8	11	8	11
Evaluation value = 0.88				
**Locus**	**Original cSCC lesion**		**KS-cSCC-2**	
TH01	7		7	
D21S11	30		30	
D5S818	9	13	13	
D13S317	8	11	8	11
D7S820	10		10	
D16S539	9	10	9	10
CSF1PO	9	12	9	
AMEL	X	Y	X	
vWA	14	17	14	17
TPOX	8	11	8	11
Evaluation value = 0.93				

### 3.3 Characteristics of the established cSCC cell lines

**Figure 2:**
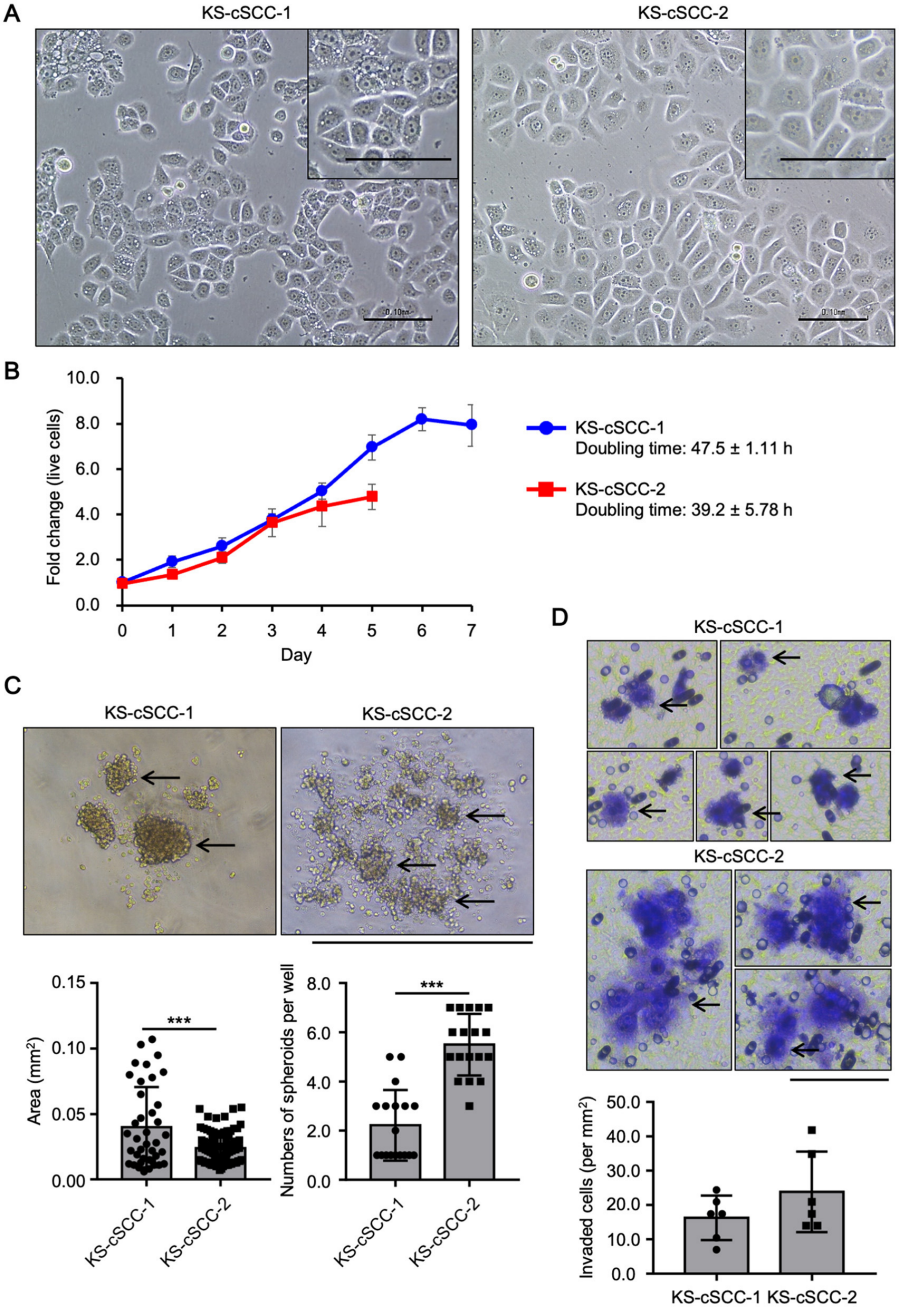
Features of newly established cSCC cell lines. **(A)** Morphology of KS-cSCC-1 and KS-cSCC-2 cells. Representative images of KS-cSCC-1 (20 passages) and KS-cSCC-2 (18 passages) cells are shown. Scale bars = 0.1 mm. **(B)** Proliferation curve of KS-cSCC-1 (15–17 passages) and KS-cSCC-2 (18–22 passages) cells. Mean ± SD of fold change of live cell number relative to that on day 0 calculated from three independent experiments is shown. **(C)** Spheroids of KS-cSCC-1 cells (23–25 passages) and KS-cSCC-2 (30–32 passages) cells. Representative images, projected area of each spheroid, and numbers of spheroids per well are shown and arrows indicate spheroid structure. Experiments were independently repeated three times with six replicate wells for each cell line. ***p < 0.001. Scale bar = 1.0 mm. **(D)** Invasion assay of KS-cSCC-1 (23 passages) and KS-cSCC-2 (32 passages) cells was performed using matrix-coated transwell culture inserts. Cells that passed through the matrix-coated membrane were stained with cell staining dye and observed. Arrows indicate invaded cells on the membrane of the culture insert. Representative images and numbers of invaded cells per unit area (mm^2^) are shown. Scale bar = 0.1 mm.

The established cell lines showed a morphology similar to that of epithelial cells and had a round nucleus with clear nucleoli (Figure 2A). The morphology of the cells was continuously observed during the cell culture period. Adherent cells were observed at 1 day after seeding and the morphology of the cells was relatively unchanged during the 9 months of the cell culture period (Supplementary Figure 3). KS-cSCC-1 and KS-cSCC-2 cell lines were composed of a homogenous cell population with the morphology of epithelial cells. Expression of vimentin, a mesenchymal marker highly expressed in fibroblasts but not in keratinocytes, was also analyzed. Since fibroblasts often contaminate the culture of patient-derived tumor cells and prevent establishment of pure tumor cell population, we removed fibroblasts by treating with lower concentration of trypsin when subculture the cells. As shown in Supplementary Figure 4, vimentin (*VIM*) expression of KS-cSCC-1 and KS-cSCC-2 cell lines was similar to that of NHEKs and A431 cells, and was significantly lower than that of fibroblasts. Considering these facts, it is estimated that the established cell lines are composed of a pure cell population and there might be very little or ignorable contamination of fibroblasts. The KS-cSCC-1 cells grew slowly, with a cell doubling time of 47.5 ± 1.11 h (Figure 2B). The KS-cSCC-2 cells tended to grow faster than KS-cSCC-1 cells, with a cell doubling time of 39.2 ± 5.78 h, although the difference was not statistically significant (*p* = 0.073, Figure 2B). Cell doubling time of the established cell lines was longer than that of NHEKs (19.0 ± 3.46 h) and A431 cells (approximately 21–26 h) (48–50). Next, spheroid formation ability (51), one of the features of tumor cells, was tested. KS-cSCC-1 formed middle to large spheroids (Figure 2C, left, arrows) when the cells were cultured in ultra-low-attachment culture plates. On the other hand, KS-cSCC-2 cells formed multiple small spheroids (Figure 2C, right, arrows). Quantification of spheroid number and the projection area showed that KS-cSCC-1 cells form significantly larger size and smaller numbers of spheroids compared to that of KS-cSCC-2 cells (Figure 2C, graphs). Invasiveness is another feature of tumor cells that is important for metastasis (52), and we also investigated it using a transwell pre-coated with matrix protein. Cells suspended in serum- and cytokine-free media were cultured on matrix-coated culture inserts and some of the KS-cSCC-1 and KS-cSCC-2 cells passed through the culture insert toward serum- and cytokine-containing media. The cells were observed on the outer side of the insert, indicating that these cell lines exhibited invasiveness (Figure 2D, arrows). Invasive cell number per unit area (mm^2^) was relatively higher in KS-cSCC-2 cells compared to KS-cSCC-1 cells, but the difference was not statistically significant (Figure 2D, graph).

### 3.4 cSCC marker expressions in the established cSCC cell lines

**Figure 3:**
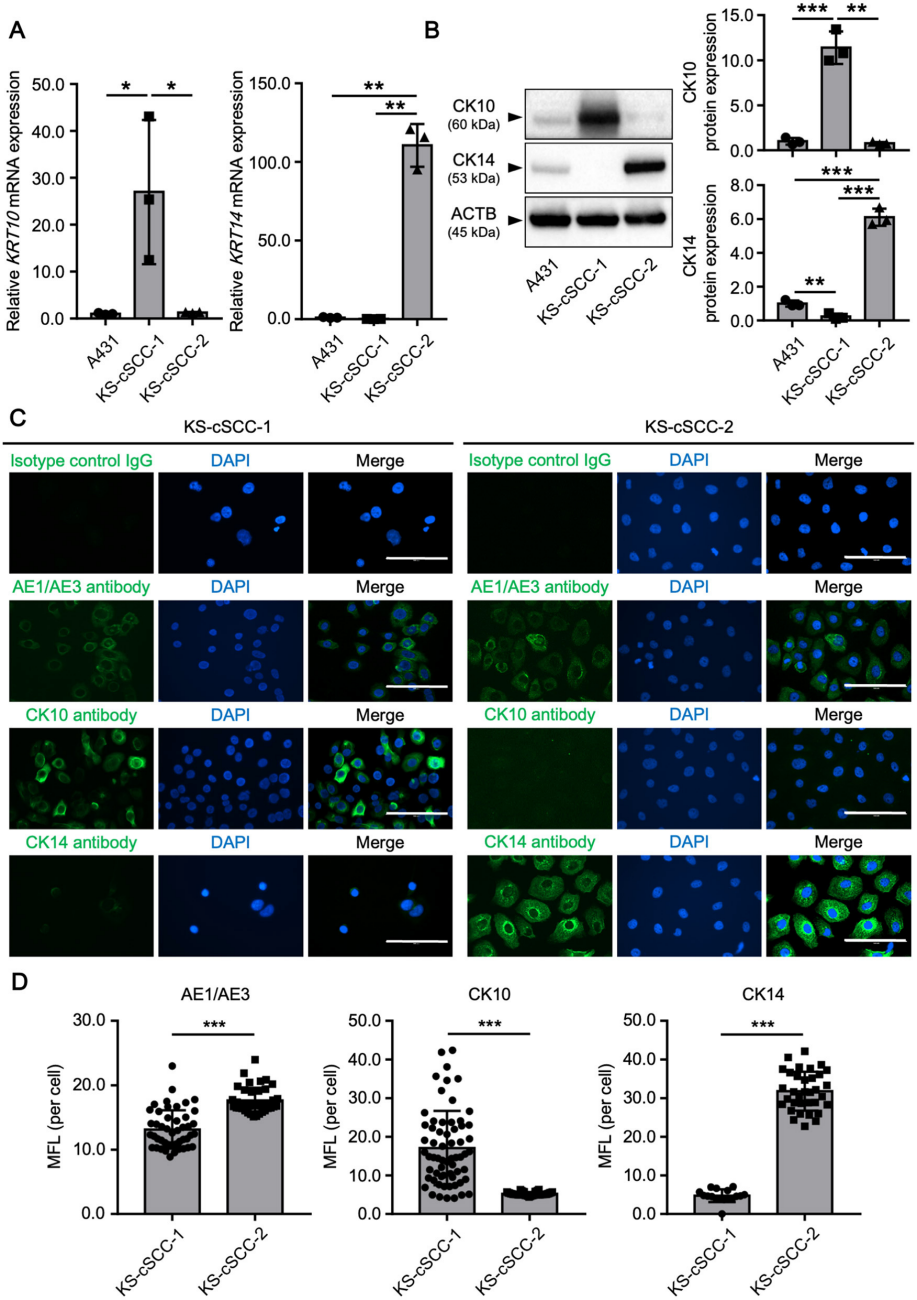
cSCC markers in newly established cSCC cell lines. **(A)** Expression of *KRT10* and *KRT14* mRNA in A431 (positive control), KS-cSCC-1 (15–17 passages), and KS-cSCC-2 (15–18 passages) cells. Mean ± SD of relative mRNA expression calculated from three independent experiments is shown. *p < 0.05 and ***p < 0.001. **(B)** Protein expression of CK10, CK14, and β-actin in A431, KS-cSCC-1 (16 passages), and KS-cSCC-2 (18 passages) cells. Representative blot images **(left)** and mean ± SD of protein expression **(right)** calculated from three independent experiments are shown. **p < 0.01 and ***p < 0.001. **(C)** Expression and localization of AE1/AE3, CK10, and CK14 proteins were analyzed in KS-cSCC-1 (15–17 passages) and in KS-cSCC-2 (15–18 passages) cells by immunocytochemistry. Experiments were independently repeated three times and representative images are shown. Scale bars = 100 μm. **(D)** Mean fluorescence intensity (MFI) of AE1/AE3, CK10, and CK14 detected by immunocytochemistry. At least three images from independent areas were used for the analysis. ***p < 0.001.

Immunohistochemical analysis of the patients' cSCC lesions showed different staining patterns of CK10 and CK14 (Figure 1). To investigate the expression of CK10 and CK14 in the established cell lines, mRNA and protein levels of CK10 (gene symbol: *KRT10*) and CK14 (gene symbol: *KRT14*) were measured. An existing SCC cell line, A431, was used for comparison. Both *KRT10* mRNA and *KRT14* mRNA were expressed in all three cell lines tested; *KRT10* was more highly expressed in KS-cSCC-1 cells than in A431 and KS-cSCC-2 cells, while *KRT14* was highly expressed in KS-cSCC-2 than in A431 and KS-cSCC-1 cells (Figure 3A). The expressions of CK10 and CK14 proteins were consistent with their mRNA levels in western blot analysis; KS-cSCC-1 showed a strong expression of CK10 and a faint to negative expression of CK14, whereas KS-cSCC-2 showed a faint CK10 expression and a strong CK14 expression (Figure 3B, Supplementary Figure 5). Protein expression and localization of cSCC markers were also assessed by immunocytochemistry. In addition to CK10 and CK14 antibodies, AE1/AE3 antibody, a cocktail antibody of pan-keratin (CK1, 3–8, 10, 12, 14–16, and 19) which detects epithelial differentiation, was used. AE1/AE3 and CK10 were strongly stained in the cytoplasm and CK14 was weakly stained in the cytoplasm of KS-cSCC-1 cells. In KS-cSCC-2 cells, AE1/AE3 and CK14 were strongly stained in the cytoplasm and CK10 was only faintly stained in the cytoplasm (Figure 3C). Mean fluorescence intensity of AE1/AE3, CK10, and CK14 was further evaluated. The intensity of AE1/AE3 and CK14 was significantly higher in KS-cSCC-2 cells compared to that in KS-cSCC-1 cells, whereas CK10 was significantly higher in KS-cSCC-1 cells (Figure 3D). Taken together, the established cSCC cell lines were confirmed to express the SCC markers with the similar patterns to their original tumor tissues.

### 3.5 Gene expression patterns in the established cSCC cell lines

**Figure 4:**
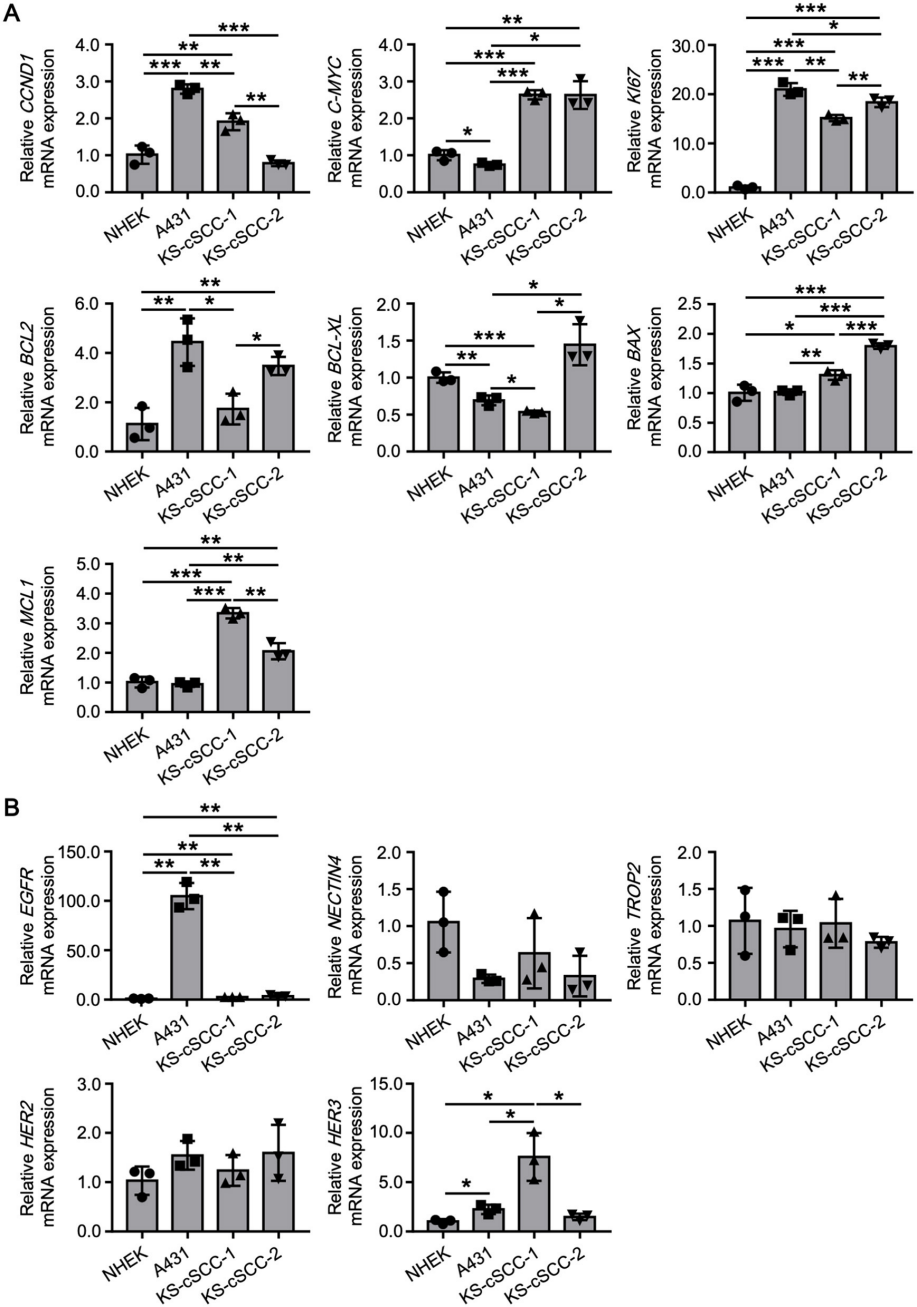
Gene expression patterns of newly established cSCC cell lines. **(A)** Expression of cell proliferation (*CCND1, C-MYC*, and *KI67*), and apoptosis and survival (*BCL2, BCL-XL, BAX*, and *MCL1*)-related genes in NHEKs (normal keratinocytes), A431 (an existing SCC cell line), KS-cSCC-1 (15–17 passages), and KS-cSCC-2 (15–18 passages) cells. Mean ± SD of relative mRNA expression calculated from three independent experiments is shown. *p < 0.05, **p < 0.01, and ***p < 0.001. **(B)** Expression of the *EGFR, NECTIN4, TROP2, HER2*, and *HER3*, which are considered to serve as therapeutic targets of cancers were assessed in NHEKs, A431, KS-cSCC-1 (15–17 passages), and KS-cSCC-2 (15–18 passages) cells. Mean ± SD of relative mRNA expression calculated from three independent experiments is shown. *p < 0.05 and **p < 0.01.

To further characterize the established cSCC cell lines, expression patterns of genes related to cell proliferation, apoptosis, and survival were assessed. NHEKs and A431 cells were also assessed to compare expression of the genes in normal and malignant keratinocytes, respectively (Figure 4A). Overexpression of cyclin D1 (*CCND1*) and *C-MYC* are frequently observed in various cancers which lead to the aberrant cell cycle and cell proliferation. *CCND1* was significantly highly expressed in A431 and KS-cSCC-1, while *C-MYC* was significantly highly expressed in KS-cSCC-1 and KS-cSCC-2, compared to that of NHEKs. The expression of *KI67*, a marker of proliferative cells, was markedly high in all three cSCC cell lines, giving more than 10-fold higher expression compared to NHEKs. Expression patterns of anti-apoptotic genes *BCL2* and *BCL-XL* were varied among each cell line. *BCL2* was significantly upregulated in A431 and KS-cSCC-2. *BCL-XL* was downregulated A431 and KS-cSCC-1, while it was upregulated in KS-cSCC-2. An anti-apoptotic gene *MCL1* and an apoptotic gene *BAX* were both significantly highly expressed in KS-cSCC-1 and KS-cSCC-2 cell lines. Additionally, expressions of *EGFR, NECTIN4, TROP2, HER2*, and *HER3*, the molecules which are considered to serve as therapeutic targets of cancers, were assessed (Figure 4B). *EGFR* was significantly upregulated KS-cSCC-1 and KS-cSCC-2 and extraordinarily high in A431 cells, more than 100-fold higher than that of NHEKs. There were no significant differences observed regarding the expression level of *NECTIN4, TROP2*, and *HER2* among 4 types of cells tested. *HER3* was significantly upregulated in A431 and KS-cSCC-1 cells compared to NHEKs.

### 3.6 Chemosensitivity against anticancer drugs

**Figure 5:**
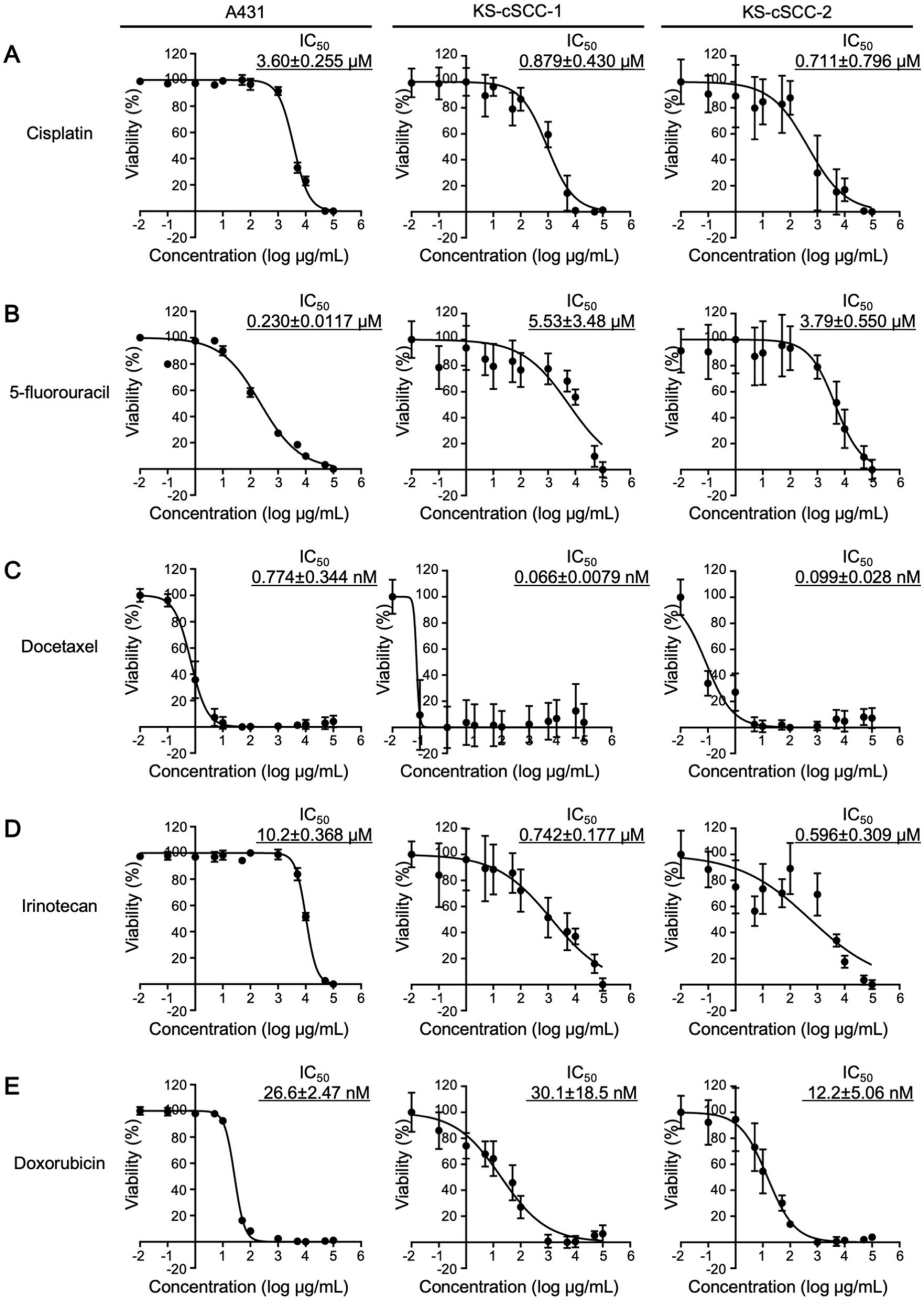
Chemosensitivity against anticancer drugs. Chemosensitivity of A431, KS-cSCC-1 (29–31 passages), and KS-cSCC-2 cell lines (30–32 passages) against **(A)** cisplatin, **(B)** 5-fluorouracil, **(C)** docetaxel, **(D)** irinotecan, and **(E)** doxorubicin was examined. Cells were treated with vehicle control (saline, DMSO, or culture-grade distilled water, 0.1%) or various concentrations of each anticancer drug (0.01 nM to 100 μM) for 72 h and viable cells were quantitated by CCK-8 assay. Experiments were performed with five wells per condition and mean ± SD of viability calculated from three independent experiments is shown.

Since the targeted therapies using cemiplimab and pembrolizumab (PD-1 inhibitors approved by the FDA) or EGFR inhibitors have not been approved in Japan, various cytotoxic anticancer drugs are used to treat cSCC that cannot be radically resected. Because standard therapies for cSCC with cytotoxic anticancer drugs have not been well established, the five anticancer drugs (i.e., cisplatin, 5-fluorouracil, docetaxel, irinotecan, and doxorubicin) have been widely used in Japan (31, 53, 54). To investigate whether the newly established cSCC cell lines are sensitive to these anticancer drugs, cells were treated with cisplatin, 5-fluorouracil, docetaxel, irinotecan, and doxorubicin and evaluated for their viability. To compare the sensitivity of the established cell lines with the existing SCC cell line, A431 cells were also treated with the anticancer drugs. The IC_50_ of each drug in A431 was calculated as follows: 3.60 ± 0.255 μM for cisplatin, 0.230 ± 0.0117 μM for 5-fluorouracil, 0.774 ± 0.344 nM for docetaxel, 10.2 ± 0.368 μM for irinotecan, and 26.6 ± 2.47 nM for doxorubicin (Figure 5). The viability of KS-cSCC-1 and KS-cSCC-2 cells was significantly decreased by all five drugs tested with various IC_50_ values (Figure 5). The IC_50_ of each drug in KS-cSCC-1 was calculated as follows: 0.879 ± 0.430 μM for cisplatin, 5.53 ± 3.48 μM for 5-fluorouracil, 0.0662 ± 0.00785 nM for docetaxel, 0.742 ± 0.177 μM for irinotecan, and 30.1 ± 1.5 nM for doxorubicin. The IC_50_ of each drug in KS-cSCC-2 was calculated as follows: 0.711 ± 0.796 μM for cisplatin, 3.79 ± 0.550 μM for 5-fluorouracil, 0.099 ± 0.028 nM for docetaxel, 0.596 ± 0.309 μM for irinotecan, and 12.2 ± 5.06 nM for doxorubicin. There were no significant differences of IC_50_ between KS-cSCC-1 and KS-cSCC-2 regarding all five drugs tested. IC_50_ of cisplatin and irinotecan was significantly higher in A431 cells compared to that in KS-cSCC-1 cells (*p* = 0.000710 and *p* = 0.00390, respectively) and in KS-cSCC-2 cells (*p* = 2.29 × 10^−6^ and *p* = 4.12 × 10^−6^, respectively). There was no significant difference of IC_50_ to docetaxel among three cell lines assessed. A431 cells showed significantly lower IC_50_ of 5-fluorouracil and higher IC_50_ of doxorubicin compared to that of KS-cSCC-2 cells (*p* = 0.00783 and *p* = 0.0115, respectively). Considering the Cmax of each drug in patient plasma (14.4 μM for cisplatin, 0.88 μM for 5-fluorouracil, 5.47 μM for docetaxel, 5.78 μM for irinotecan, and 6.78 μM for doxorubicin) (55), KS-cSCC-1 and KS-cSCC-2 cells are highly sensitive to these drugs and show distinct sensitivity when compared with A431 cells.

## 4 Discussion

Despite the overall favorable prognosis and relative rarity of mortality among affected individuals, cSCC is associated with a high total number of deaths due to its high incidence (1, 56). Although anti-PD-1 therapy has prolonged the survival of patients with advanced cSCC, there is still an urgent need to explore second-line treatments. In the current study, we established the KS-cSCC-1 and KS-cSCC-2 cell lines from patients' lymph node metastases as the resources for basic study on cSCC.

On immunohistochemistry, both the metastatic tumor tissues were positive for p63 and AE1/AE3, which is well concordant with the expression pattern of cSCC. Interestingly, the KS-cSCC-1 cells exhibited strong expression of CK10 and weak expression of CK14 in western blotting and immunocytochemistry. However, the original metastatic tumor showed only focal staining for CK10 and CK14 on immunohistochemistry. On the other hand, the KS-cSCC-2 cells strongly expressed CK14 and negatively-to-faintly expressed CK10, which is in sharp contrast to KS-cSCC-1. These suggest that KS-cSCC-1 was derived from a CK10^+++^CK14^+^ tumor cell and KS-cSCC-2 from CK10^−^CK14^+++^ tumor cell in these heterogeneous cancer tissues. Keratin expression pattern of the KS-cSCC-1 cell line contrasts markedly with that of the A431 and KS-cSCC-2 cell lines. Although the A431 cell line is widely used as a model of SCC, it originates from an epidermoid carcinoma of the vulva (38–40) and is often used to represent the vaginal mucosa (57). In addition, cSCC is frequently found in the sun-exposed area since ultra-violet (UV) exposure is the main causal risk factor of this disease (58), whereas A431 is derived from non-sun-exposed area. Thus, it should be distinguished from cutaneous SCC in a strict sense. Indeed, differences between A431 and the newly established cell lines were observed in the expressions of multiple molecules related to cancer (Figure 4). From another point of view, A431 cell line is often used to study EGFR since EGFR is overexpressed in this cell line. It is reported that EGFR was detected in 90% of cSCC cases but EGFR overexpression was observed in 35% (59), indicating that it does not represent the majority of this tumor. These results and knowledges suggest that the combined use of A431, KS-cSCC-1, and KS-cSCC-2 may provide a good representation of cSCC, as a highly heterogeneous cancer.

As noted above, cSCC is a highly heterogeneous tumor. Indeed, there were obvious intra- and inter-tumor heterogeneities observed in our patient's tumor lesion (Figure 1, Supplementary Figures 1, 2). It is reported that sun-exposure changes expression of hundreds of genes which may contribute to the development of cSCC (60, 61). Thus, attention needs to be paid to interpret the data obtained from the experiments using cell lines. It is ideal to use multiple cSCC cell lines with various backgrounds. However, most of the existing SCC cell lines except for A431 were established by each institute and it is difficult to obtain their detailed characteristics to compare with the newly established cell lines. Thus, we here used A431, a representative SCC cell line that is commercially available and is widely used for investigations. As shown in several figures, A431 and newly established cell lines showed different characteristics especially gene expression and chemosensitivity. From the point of view of drug development and personalized medicine, these differences will give useful information to screen therapeutic targets for cSCC. We understand that comparison with other cell lines is also important to obtain more detailed insights and it will be performed in the future studies.

Existence of sex differences has been known in various cancers. It is thought that multiple factors such as genotoxic stress and DNA damage response, X chromosome, antitumor immunity, hormones, epigenetics, and metabolisms may cause the differences between males and females, which may eventually affect treatment and prognosis of the patients (62). Until now, cSCC is thought to have less sex differences and little is known about it. There are some reports showing that males have higher incidence of cSCC compared to that of females (63, 64). It has been suggested that occupational and lifestyle reasons which affect exposure to sunlight may cause the difference, however, it still lacks objective indicators of the relationships. Gender differences in UVB-induced skin cancer have been reported using a Skh-1 mouse model (65). In the model, earlier tumor development, larger and more tumor formation, and greater total tumor burden was found in male mice compared to females, although the clear mechanisms which caused the difference remain unknown. Another mouse model focusing on the effect of hormone (i.e., estrogen) suggested the protective role of estrogen against skin tumorigenesis partly through inhibiting cyclin D1 (66). Although the role of estrogen and its receptor was also reported in a human SCC cell line A431, the activation of estrogen receptor in A431 cells upregulated cyclin D1, contrary to what was observed in mice (64). Thus, the sex differences of cSCC still remain unclear and it is hard to judge whether the different characters observed in the newly established cell lines represent sex differences or just the individual differences. In this case, KS-cSCC-1 (derived from a female) and KS-cSCC-2 (derived from a male) cell lines might be applied for the research to investigate sex differences in cSCC through performing genetic analysis and molecular analyses.

Before the era of immunotherapy, cytotoxic chemotherapy was frequently used for metastatic cSCC. On chemosensitivity tests, cisplatin, 5-fluorouracil, docetaxel, irinotecan, and doxorubicin, which are key chemotherapy drugs for cSCC, reduced the tumor cell viability of KS-cSCC-1 and KS-cSCC-2, suggesting that these cell lines can be used as the *in vitro* models for cSCC. Of note, although the IC_50_ to each drug was not different between KS-cSCC-1 and KS-cSCC-2 cells, the value was distinct from that of A431 cells, an existing SCC cell line which has been used experientially (Figure 5). Since the background of the A431 cell line is different from KS-cSCC-1 and KS-cSCC-2 cells as mentioned above, use of the newly established cell lines will give multifaceted information on development of anticancer drugs to cSCC. One of the limitations of this study is that more comprehensive genetic analyses (whole-exome sequencing, etc.) to compare patient tissues and the established cell lines could not be performed; specifically, the quality of DNA extracted from the patients' tissues were low due to the abundant necrotic tissues. Identity between the patient tumors and the cell lines was further validated by STR analysis and confirmed that the cell lines are identical to their original tumors. Comprehensive genetic characterizations as well as molecular profiles using the established cell lines will be addressed in the future studies to further characterize the established cell lines and to get some insights into the utility of the cell lines compared to the other existing cSCC cell lines. At present, a cancer multi-gene panel testing of the female patient (source of KS-cSCC-1) revealed abnormalities in *BAP1, CDKN2A, CDKN2B, MET, MTAP, MYC, RAD21, STK11*, and *TP53* genes, attentions will be paid to those genes first. Another limitation of this work is lack of the *in vivo* xenograft models using immunodeficient mice to confirm tumor-forming ability and to use for preclinical studies. This will be also addressed in the future studies when the assay system is established.

## 5 Conclusion

In summary, we have established two novel cell lines, KS-cSCC-1 and KS-cSCC-2. Since cSCC cell lines from Japanese patients are rare, KS-cSCC-1 and KS-cSCC-2 cell lines should be useful for basic and preclinical research to obtain a better understanding of this heterogenous tumor.

## Data Availability

The original contributions presented in the study are included in the article/Supplementary material, further inquiries can be directed to the corresponding author.
