# Supplementary material for: KS-cSCC-1 and KS-cSCC-2: two novel cutaneous squamous cell carcinoma cell lines established from Japanese patients
Source: Front Med (Lausanne). 2024 Nov 7;11:1483450. doi: 10.3389/fmed.2024.1483450 (PMC11583063; doi:10.3389/fmed.2024.1483450)
Supplement: Supplementary file 1 [file Data_Sheet_1.docx]

Supplementary Material

##
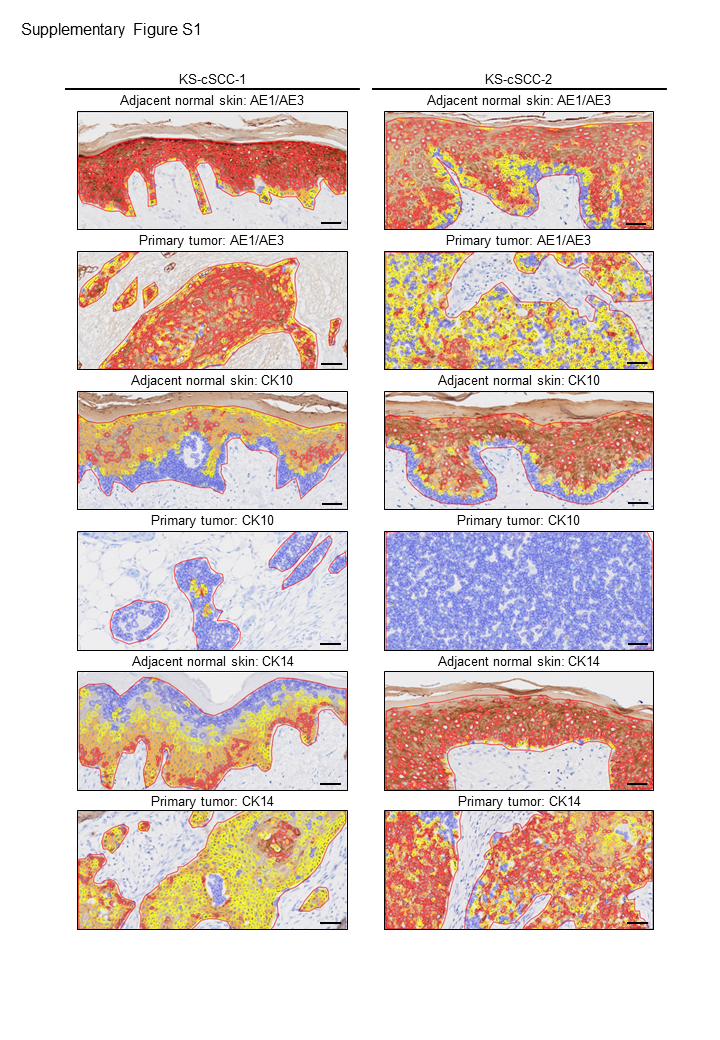
 Supplementary Figure 1

**Supplementary Figure 1.** Analysis of AE1/AE3, CK10, and CK14 staining using QuPath in adjacent normal skin and primary tumors of KS-cSCC-1 and KS-cSCC-2. Scale bars = 50 μm.

##
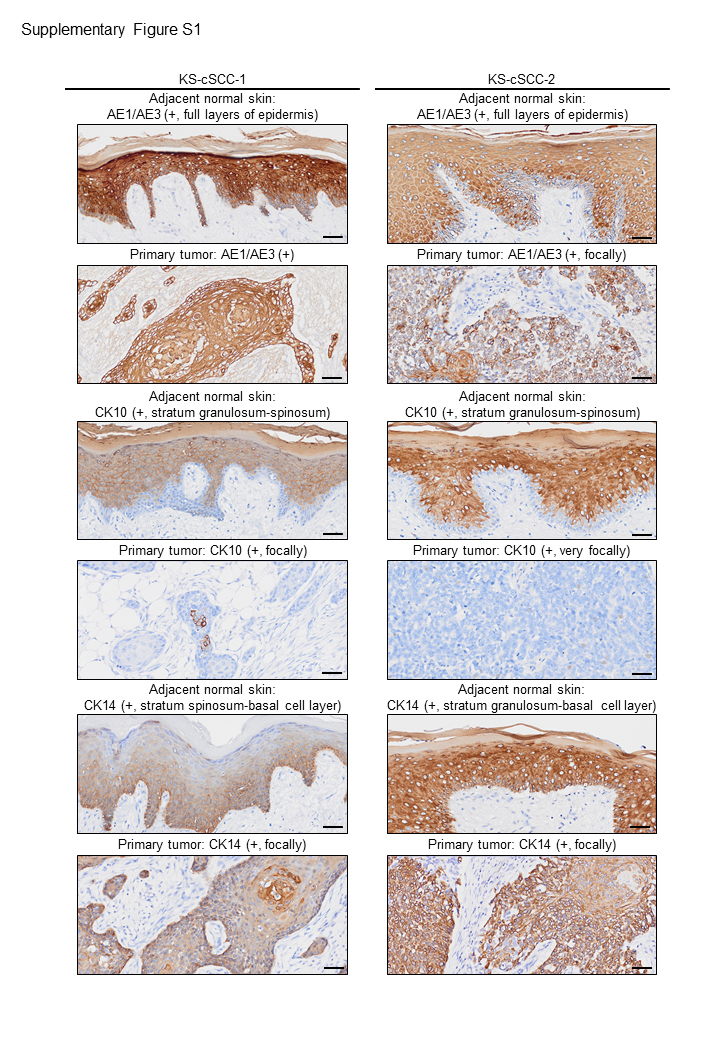
Supplementary Figure 2

**Supplementary Figure 2.** IHC of AE1/AE3, CK10, and CK14 in adjacent normal skin and primary tumors of KS-cSCC-1 and KS-cSCC-2. Scale bars = 50 μm.

## Supplementary Figure
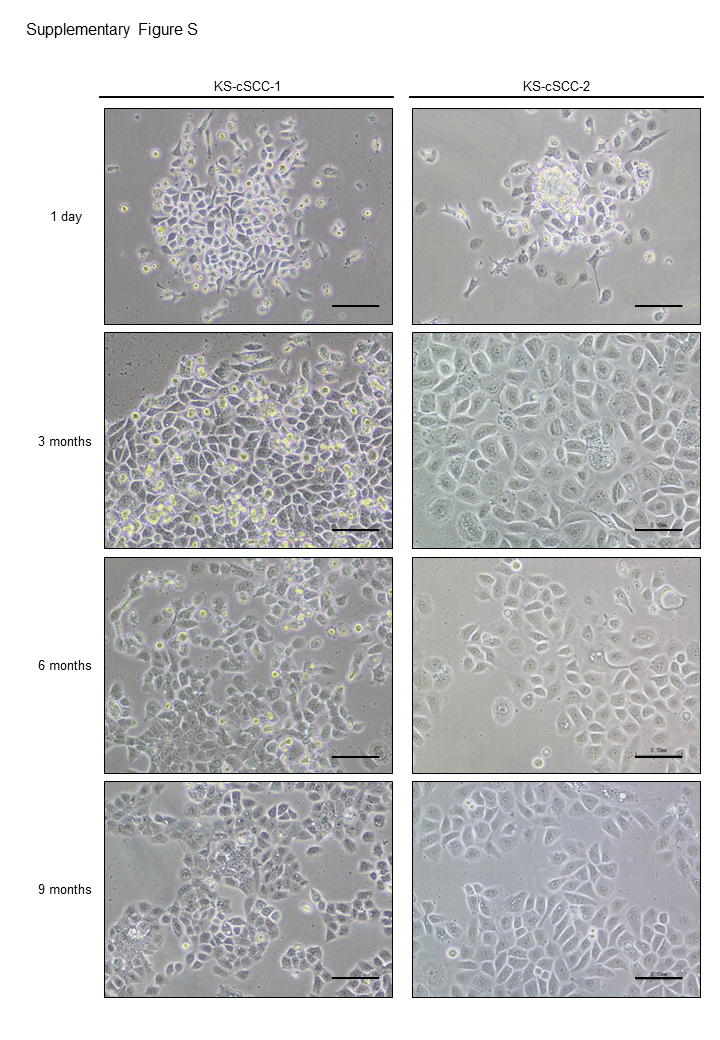
3

**Supplementary Figure 3.** Morphology of KS-cSCC-1 and KS-cSCC-2 cells during 9 months of culture period. Scale bars = 0.1 mm.

## Supplementary Figure 4


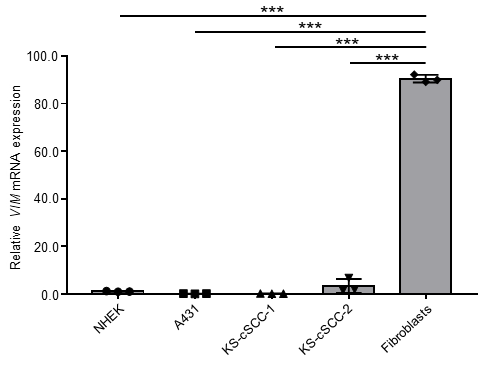


## Supplementary Figure 4. Expression of vimentin mRNA (*VIM*) in NHEKs (normal keratinocytes), A431 (an existing SCC cell line), KS-cSCC-1 (15–17 passages), and KS-cSCC-2 (15–18 passages), and fibroblasts (normal dermal fibroblasts). Mean ± SD of relative mRNA expression calculated from three independent experiments is shown. ****p* < 0.001.

## Supplementary Figure 5

**
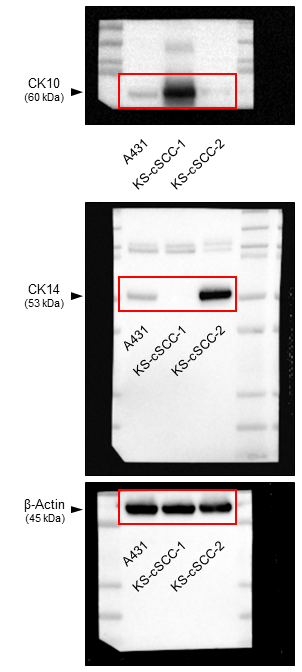
**

## Supplementary Figure 5. Full-length blots presented in Fig. 3B. CK10, CK14, and β-Actin protein expression in A431, KS-cSCC-1, and KS-cSCC-2 cells was determined by western blotting. Unedited original images of blots are shown. The red boxes indicate the cropped areas shown in Fig. 3B.

## Supplementary Table 1. Sequences of primers used for qRT-PCR.

| Gene symbol | Primer sequence |
| --- | --- |
| *ACTB* | Sense: 5′-ATTGCCGACAGGATGCAGA-3′ |
|  | Antisense: 5′-GAGTACTTGCGCTCAGGAGGA-3′ |
| *BAX* | Sense: 5′-GGACGAACTGGACAGTAACATGG-3′ |
|  | Antisense: 5′-GCAAAGTAGAAAAGGGCGACAAC-3′ |
| *BCL2* | Sense: 5′-ATCGCCCTGTGGATGACTGAG-3′ |
|  | Antisense: 5′-CAGCCAGGAGAAATCAAACAGAGG-3′ |
| *BCL-XL* | Sense: 5′-TTACCTGAATGACCACCTA-3′ |
|  | Antisense: 5′-ATTTCCGACTGAAGAGTGA-3′ |
| *CCND1* | Sense: 5′-GCTGCGAAGTGGAAACCATC-3′ |
|  | Antisense: 5′-CCTCCTTCTGCACACATTTGAA-3′ |
| *CK10*  (*KRT10*) | Sense: 5′-CCTGCTTCAGATCGACAATGCC-3’ |
|  | Antisense: 5′-ATCTCCAGGTCAGCCTTGGTCA-3’ |
| *CK14*  (*KRT14*) | Sense: 5′-TGCCGAGGAATGGTTCTTCACC-3’ |
|  | Antisense: 5′-GCAGCTCAATCTCCAGGTTCTG-3’ |
| *C-MYC* | Sense: 5′-GAGGCGAACACACAACGTCTT-3′ |
|  | Antisense: 5′-CGCAACAAGTCCTCTTCAGAAA-3′ |
| *EGFR* | Sense: 5′-AACACCCTGGTCTGGAAGTACG-3’ |
|  | Antisense: 5′-TCGTTGGACAGCCTTCAAGACC-3’ |
| *HER2* | Sense: 5′-GGAAGTACACGATGCGGAGACT-3’ |
|  | Antisense: 5′-ACCTTCCTCAGCTCCGTCTCTT-3’ |
| *HER3* | Sense: 5′-CTATGAGGCGATACTTGGAACGG-3’ |
|  | Antisense: 5′-GCACAGTTCCAAAGACACCCGA-3’ |
| *KI67* | Sense: 5′-TTGGAGAATGACTCGTGAGC-3’ |
|  | Antisense: 5′-CGAAGCTTTCAATGACAGGA-3’ |
| *MCL1* | Sense: 5′-AACAAAGAGGCTGGGATG-3’ |
|  | Antisense: 5′-ATTGCACTTACAGTAAGGCTATC-3’ |
| *NECTIN4* | Sense: 5′-CAAAATCTGTGGCACATTGG-3’ |
|  | Antisense: 5′-GCTGACATGGCAGACGTAGA-3’ |
| *TROP2* | Sense: 5′-CCTCATCGCCGTCATCG-3′ |
|  | Antisense: 5′-CGGTTCCTTTCTCAACTCCC-3′ |
| *VIM* | Sense: 5′-GAGAACTTTGCCGTTGAAGC-3′ |
|  | Antisense: 5′-GCTTCCTGTAGGTGGCAATC-3′ |
